# Protein expression-independent response of intensity-based pH-sensitive fluorophores in *Escherichia coli*

**DOI:** 10.1371/journal.pone.0234849

**Published:** 2020-06-18

**Authors:** Kathy Y. Rhee, Ravi Chawla, Pushkar P. Lele

**Affiliations:** Artie McFerrin Department of Chemical Engineering, Texas A&M University, College Station, Texas, United States of America; University of California Irvine, UNITED STATES

## Abstract

Fluorescent proteins that modulate their emission intensities when protonated serve as excellent probes of the cytosolic pH. Since the total fluorescence output fluctuates significantly due to variations in the fluorophore levels in cells, eliminating the dependence of the signal on protein concentration is crucial. This is typically accomplished with the aid of ratiometric fluorescent proteins such as pHluorin. However, pHluorin is excited by blue light, which can complicate pH measurements by adversely impacting bacterial physiology. Here, we characterized the response of intensity-based, pH-sensitive fluorescent proteins that excite at longer wavelengths where the blue light effect is diminished. The pH-response was interpreted in terms of an analytical model that assumed two emission states for each fluorophore: a low intensity protonated state and a high intensity deprotonated state. The model suggested a scaling to eliminate the dependence of the signal on the expression levels as well as on the illumination and photon-detection settings. Experiments successfully confirmed the scaling predictions. Thus, the internal pH can be readily determined with intensity-based fluorophores with appropriate calibrations irrespective of the fluorophore concentration and the signal acquisition setup. The framework developed in this work improves the robustness of intensity-based fluorophores for internal pH measurements in *E*. *coli*, potentially extending their applications.

## Introduction

Bacteria maintain a tight control over their cytoplasmic pH even when the extracellular pH fluctuates significantly [1–5]. Homeostasis in the cytoplasmic pH is crucial for the regulation of important processes including enzymatic function, metabolism, ion channel activity, motility, and cell division [6–8]. Homeostasis is promoted by several intrinsic buffering mechanisms including those that involve enzymatic systems including amino acid decarboxylases [9–13], transcription factors [14, 15], ammonia-producing deaminase and deiminase systems [16], and ureases [17, 18]. Other mechanisms limit proton transport by modulating proton permeability of the phospholipid membranes and proton antiporter activity [10, 12, 19]. Gram-negative *Escherichia coli* are remarkable at maintaining pH homeostasis; their cytoplasmic pH ranges narrowly between 7.4–7.8 [6, 20, 21].

Intracellular pH has been traditionally measured with the aid of extracellular probes whose uptake by the cells is pH-dependent. Examples are membrane-permeant radiolabeled probes and fluorescent dyes [20, 22–24]. However, these approaches afford relatively low temporal and spatial resolution. Cell-cell variability in probe (or dye) uptake and leakage limit the accuracy of measurements [25]. The use of pH-sensitive fluorescent proteins helps circumvent these problems as the proteins are expressed intracellularly.

Several variants of the green fluorescent protein (GFP) have been developed that vary their emission intensities as a function of the ambient pH [26]. These so-called intensity-based probes exhibit two states that differ in the protonation state of the chromophore and that have distinct spectral characteristics [27]. Changes in the pH induce a shift in the equilibrium between the two states, thereby modulating the emission [28, 29]. Fluorescent proteins have become popular alternatives to traditional pH probes [30–32]. They have been widely employed in combination with spectroscopy, flow cytometry, and fluorescence microscopy [33, 34] to measure relative changes in pH with superior temporal and spatial resolution [35, 36].

The fluorescence signal is proportional to the number of fluorescent proteins expressed within each cell. In bacteria, cell-cell variability in the number of fluorophores can cause large fluctuations in the signal from intensity-based probes, especially given the tiny cell volumes. Fluorophore expression levels may also vary significantly across different mutant strains or growth conditions, complicating the quantification of cytosolic pH. To overcome this limitation of intensity-based probes, ratiometric fluorescent proteins such as pHluorin, GFpH, Rosella, and deGFP have been developed [37–40]. Emissions are obtained from such probes under different excitation or emission settings, and the ratios of the signals are determined since they are independent of the expression levels of the fluorophores [41].

A key limitation in the use of the popular ratiometric probe, pHluorin, is that the illumination wavelengths used to excite the fluorophore promote the well-known blue light effect–incident light at 405 nm has bactericidal effects on a variety of species [42–46]. This could interfere with pH measurements by affecting cell physiology [45, 47]. Another problem is the need for multi-wavelength excitation setups, which can cost more and can limit measurements of rapid changes in the pH, especially if the excitation wavelength must be repeatedly alternated.

In this work, we explored whether pH-sensitive emissions from existing intensity-based fluorescent proteins can provide similar advantages as the ratiometric probes. We showed that the emissions from Gfpmut3* and eYFP, two probes that excite at wavelengths where the blue light effect is diminished, can be made independent of the expression levels with a simple scaling analysis. The scaled signals were also independent of the photon excitation and detection settings. This suggests that intensity-based probes could be employed to reliably detect internal pH when conditions do not favor the use of ratiometric proteins.

## Materials and methods

### Cell culturing

All strains were derived from *E*. *coli* RP437 [48]. PCR-amplified fluorescent protein alleles were inserted into the MCS (multiple cloning sites) region of the *pTrc99A* vector to generate desired plasmids: pTrc99A-Gfpmut3*, *pTrc99A-eyfp*, and *pTrc99A-pHluorin*. DsRed-Express (*pUC19* vector backbone) was obtained from Clontech (Cat. No. 632412). Overnight cultures were grown from isolated colonies in Tryptone broth (TB). Day cultures were grown from the overnight cultures (1:100 dilution) in 10 mL of fresh TB at 33°C. Ampicillin was added at a final concentration of 100 μg/mL to the overnight and day cultures. Expression levels of the fluorophores were controlled by adding 0–100 μM isopropyl β-D-1-thiogalactopyranoside (IPTG) to the day culture at the time of inoculation. The cultures were grown to an OD_600_ ~ 0.5 before washing three times via centrifugation (1500g, 5 min) in phosphate buffer (0.01M Phosphate buffer, 0.067 M NaCl, 10^-4^ M EDTA, 0.01M Sodium Lactate and 1 μM Methionine) [49, 50].

### Modulation of intracellular pH

To measure changes in fluorophore emission intensities at varying pH, tunnel slides were prepared by sticking two glass surfaces together with double-sided adhesive tapes. Approximately 30 μl of 0.01% poly-L-lysine solution was introduced in the tunnel slide for ~ 5 min. It was subsequently exchanged with DI water or buffer by gently wicking the fluid from one end of the tunnel slide and adding ~ 7 times higher volume of the replacement fluid at the other end. This minimized the contact of the cells with poly-L-lysine. A concentrated cell suspension was then introduced and was allowed to stand for ~ 10 min to enable the cells to sediment and adhere to the coverslip. The unstuck cells were gently removed by wicking the fluid while adding buffer at the other end. The stuck cells were exposed to buffers of desired pH (5.0 to 9.0) containing 40 mM benzoate [33, 36, 51] for at least 5–10 min prior to the measurements.

### Fluorescence assays

**Fig 1:**
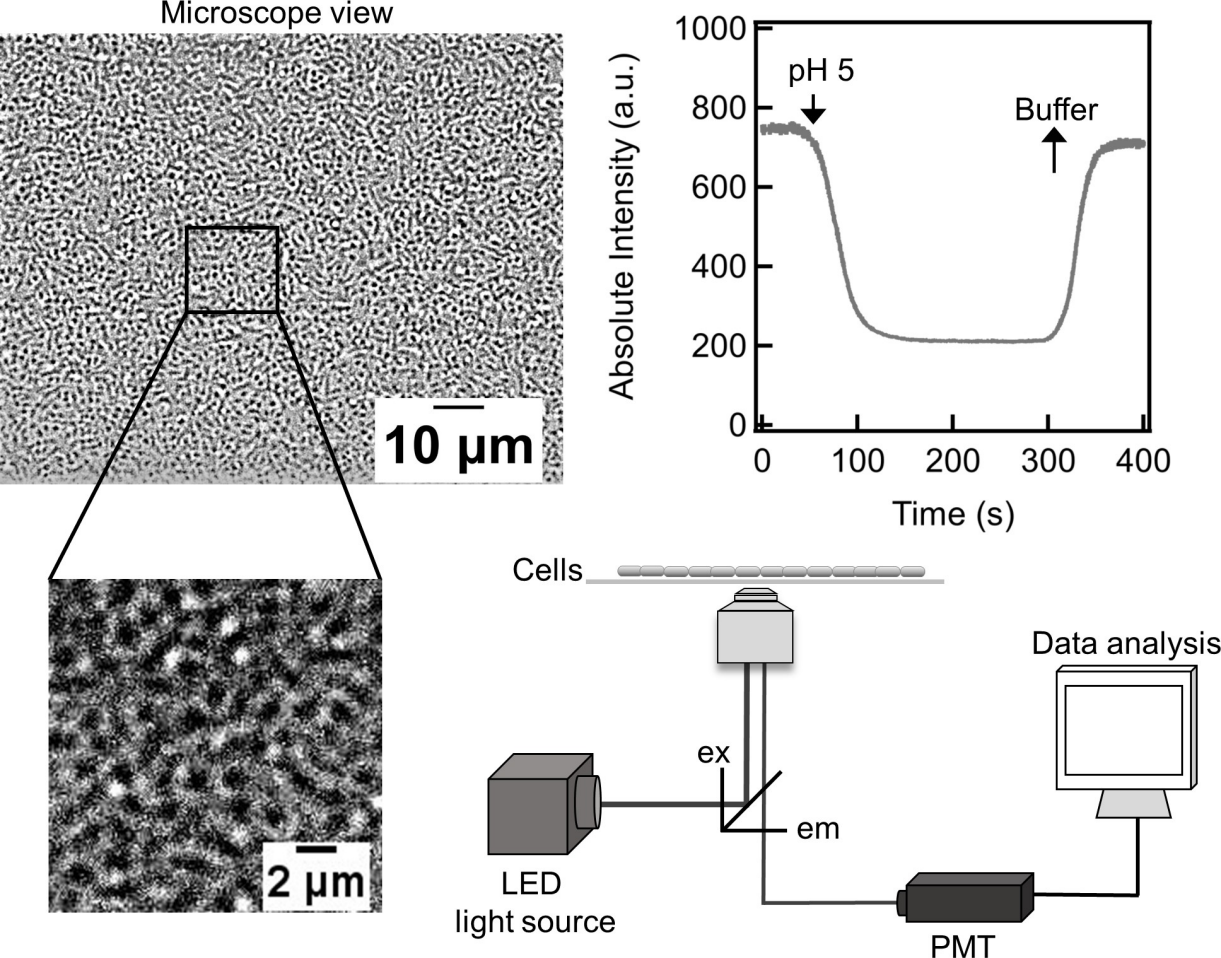
Schematic of the experimental set-up. A typical region of observation is shown (top left); inset shows a closer view of several cells stuck to the coverslip. White LED light was passed through a suitable excitation filter (ex) before illuminating the sample. The emissions were filtered with an emission filter (em) and relayed to a photon-counting photomultiplier (bottom right). Time-varying emissions from Gfpmut3*-containing cells is shown (top right). The neutral buffer was exchanged with an acidic medium containing 40 mM benzoate at ~ 50 s. A sharp reduction in intensity was observed in real-time. The original buffer was re-introduced at ~ 300 s, following which the intensity returned to its pre-stimulus value (after accounting for photobleaching). The calculated statistical power for the dynamic response was 1.

The live cells were imaged on a Nikon Ti-E microscope with a 60x water immersion objective (Nikon Instruments). The coverslip was first scanned to select a region where the cell-coverage was dense and uniform (Fig 1); regions with vacant areas were ignored. Then, an LED illumination source (SOLA SE II 365 light engine, Lumencor) was used to excite the fluorophores. Emissions were collected from ~1000 cells at any instant (Fig 1). Background correction was not deemed necessary since the coverslip contributed less than 4% to the overall emission. Excitation and the emission signals were appropriately filtered depending on the fluorescent protein (see Table 1). The emissions were relayed to a sensitive photomultiplier (H7421-40 SEL, Hamamatsu Corporation) and the photon-counts were recorded with custom-written LabView codes at a sample rate of 10 Hz for ~ 30 seconds. For pHluorin, 15 seconds of data was recorded for each excitation wavelength: 410 nm and 475 nm. The illumination intensities from the light engine were attenuated such that the emissions stayed within the dynamic range of the photomultiplier. For each tunnel slide, emissions were recorded from four different regions. For each fluorophore, three biological replicates were carried out. For the representative pH-response shown in Fig 1, a perfusion chamber was employed, which enabled media to be exchanged.

**Table 1:** Filter information.

Fluorescent protein	Excitation	Emission
eYFP	Zet514/10x^†^	FF01-542/27*
Gfpmut3*	FF01-466/40x*	FF03-525/50x*
pHluorin	FF01-466/40x* FF01-409/32-25*	FF03-525/50x*
DsRed-Express	FF01-542/22*	FF01-598/25*

†—Chroma Tech Corp.

*- Semrock Inc.

### Statistical testing

Two-tailed paired t-test was employed to determine statistical significance. Difference in mean intensities was considered significant for p < 0.05.

## Results

### pH-sensitive fluorophore emission

We separately expressed Gfpmut3*, eYFP, and pHluorin in *E*. *coli* from a common, inducible expression vector. Cells were stuck to poly-L-lysine coated coverslips in tunnel slides and their fluorescence emissions were measured with a photomultiplier. To measure emissions over a range of intracellular pH, cells were treated with 40 mM benzoate in phosphate buffers maintained at specific pH values (see Materials and methods). In its protonated form, benzoate permeates the membrane and equalizes the cytoplasmic and extracellular pH [33, 36].

**Fig 2:**
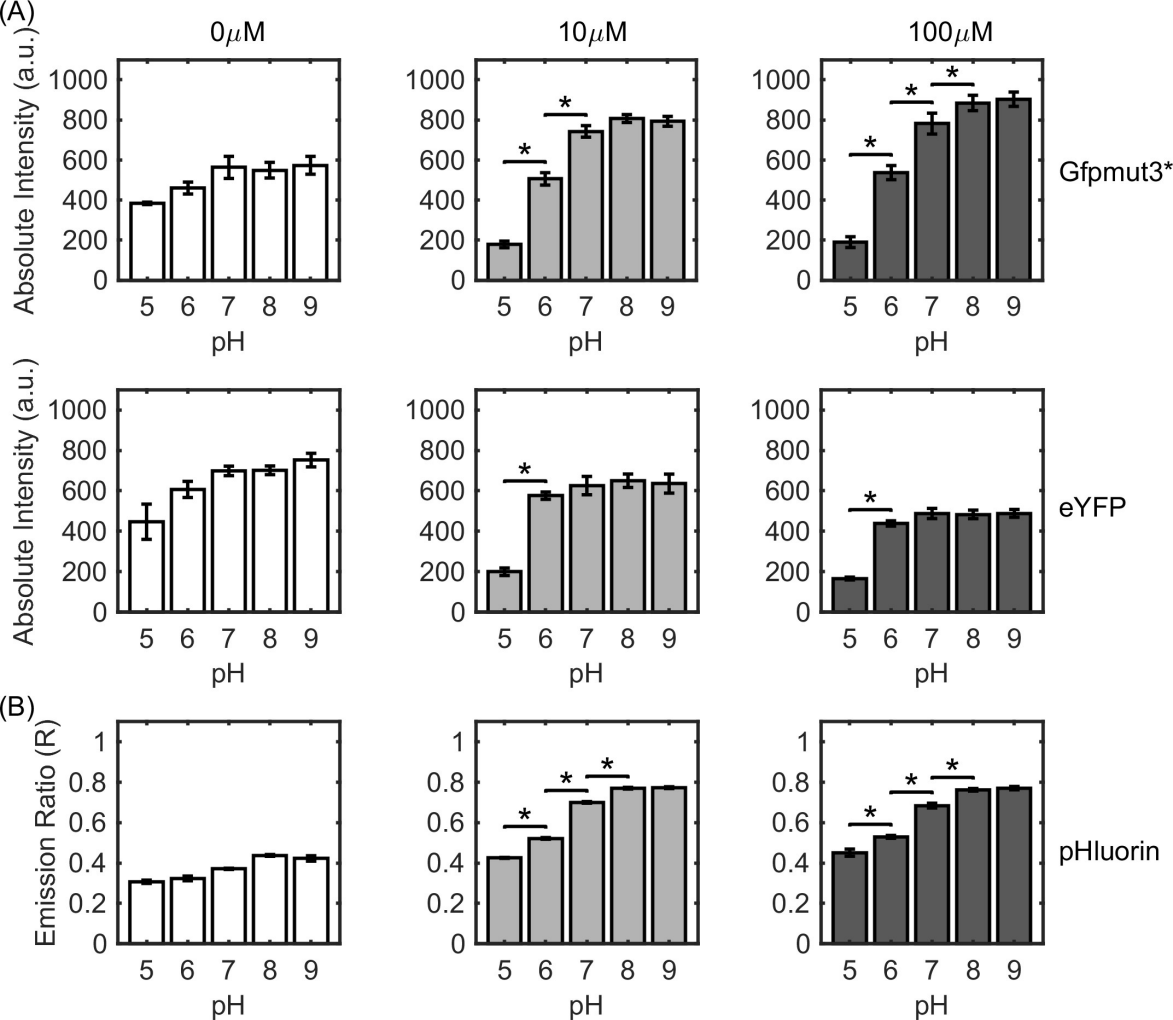
pH-sensitive emission intensities for fluorophores. **(A)** Absolute intensities are indicated over a range of cytoplasmic pH values for Gfpmut3* (top row) and eYFP (bottom row). **(B)** The ratios of emissions at two excitation wavelengths for pHluorin are shown as a function of cytoplasmic pH. IPTG concentrations (induction levels) were 0 μM (white bars), 10 μM (light gray), and 100 μM (dark gray). Since the absolute intensities for all three fluorophores increased with the induction levels (S1 Fig), the illumination intensity was attenuated as needed to operate within the dynamic range of our photomultiplier. Each data point reflects the mean value determined from four technical and three biological replicates (n ~ 12,000 cells). Standard error was estimated from the biological replicates; * indicates p-value < 0.05.

Gfpmut3* and eYFP are intensity-based fluorophores, where the excitations and emissions of interest occur over single wavelengths (Table 1). The absolute emission intensities of Gfpmut3* and eYFP over varying pH are shown in Fig 2A. The intensities were the lowest at pH 5.0 and increased with pH before plateauing for pH values > 7.0. Three induction levels were tested for each type of fluorophore: basal or low expression (0 μM IPTG), medium expression (10 μM IPTG) and high expression levels (100 μM IPTG). The emission intensities increased with protein expression levels (S1 Fig). Hence, the excitation was appropriately attenuated to work within the dynamic range (operating limits) of our photomultiplier detection system. The fluorophores exhibited similar responses over varying internal pH at different expression levels.

pHluorin displays a dual excitation peak at 410 nm and 475 nm with a common emission at a wavelength of 509 nm [37]. Therefore, two excitation wavelengths were employed to measure the pH-sensitive signals from this fluorophore. The emission ratio was calculated by dividing the emissions at the lower excitation wavelength by the emissions at the higher excitation wavelength. The emission ratios versus internal pH for pHluorin are shown in Fig 2B for the three expression levels. The dependence of the ratios on the pH was qualitatively similar to the emission curves obtained for Gfpmut3* and eYFP. At the highest induction level studied, pH-induced changes in the signals of Gfpmut3* (and of pHluorin) were statistically significant until pH 8; changes in the signals of eYFP were significant until pH 6. This suggests that Gfpmut3* is effective in detecting unit change in pH over the range of pH 5 to 8. At the basal expression level (0 μM IPTG), pH-induced changes in the signals were not significant for any of the three fluorophores.

In contrast to the three pH-probes, a pH-insensitive protein, DsRed-Express, exhibited insignificant variations in emission over the pH range studied here (S2 Fig), in agreement with prior reports [52].

### Analytical model to eliminate dependence of signals on expression levels

For intensity-based pH probes, the total emission intensity (*I*^*cell*^) from each cell can be represented as a sum of the emissions from the protonated, *N*_*prot*_, and the deprotonated, (*N–N*_*prot*_), subpopulations.

$$I^{cell}={(N-N}_{prot})I_{deprot}+N_{prot}I_{prot}$$ (1)

*N* is the total fluorophore population. *I*_*prot*_ and *I*_*deprot*_ refer to the emission intensities of individual protonated and the deprotonated fluorophores. The emission from the protonated fluorophore is lower than the deprotonated fluorophore (*I*_*prot*_ *< I*_*deprot*_). We assume that there are no interactions between the fluorophores.

The fraction of the protonated fluorophores within the cell is assumed to be in equilibrium with the proton abundances within the cell, [*H*^*+*^]_*in*_:

$$\frac{N_{prot}}{N}=\frac{{\left[H^{+}\right]}_{in}}{K+{\left[H^{+}\right]}_{in}}$$ (2)

Here, *K* is the dissociation constant which depends on the type of the fluorophore. Eq 2 is a reasonable assumption since the cell has a considerable buffering capacity and carries millions of ionizable groups that are more abundant than the fluorophores. The proton abundance was estimated from the internal pH as per the relation:

$${\left[H^{+}\right]}_{in}=10^{-pH}*N_{A}*V_{E.\;coli}$$ (3)

*N*_*A*_ is the Avogadro’s number, and *V*_*E*.*coli*_ is the cytoplasmic volume of *E*. *coli*.

As is evident from Eqs 1–3, the signal from each cell is dependent on the fluorophore intensities (*I*_*prot*_ and *I*_*deprot*_), the fluorophore expression levels (*N*), and *K*. A key outcome from the model is that when the intensity is scaled (min-max normalization), the scaled intensity (*Î*) becomes independent of the emission intensities altogether (see S1 Appendix):

$${\hat{I}}^{cell}=\frac{I^{cell}-I_{min}^{cell}}{I_{max}^{cell}-I_{min}^{cell}}=\frac{\left(N_{prot}^{pH\;min}-N_{prot}^{pH}\right)}{\left(N_{prot}^{pH\;min}-N_{prot}^{pH\;max}\right)}$$ (4)

This is convenient since it theoretically eliminates variations associated with the differences in illumination sources, detector gain and filters among other factors.

The scaled or normalized intensity at a given pH can be further shown to depend solely on *K* and the minimum pH value employed in the normalization. Since the difference in the maximum and minimum pH values chosen in our work is large (9 and 5, respectively), the condition $N_{prot}^{pH\;min}\gg N_{prot}^{pH\;max}$ is satisfied for most fluorescent proteins (pKa values ≲ 7.0).

$$∴{\hat{I}}^{cell}∼1-\frac{\left(1+\frac{K}{{\left[H^{+}\right]}_{in}^{pH\;min}}\right)}{\left(1+\frac{K}{{\left[H^{+}\right]}_{in}}\right)}$$ (5)

Eq 5 indicates that the scaled intensities are independent of the expression levels of intensity-based fluorescent proteins, such as GFP and YFP. Finally, it is straightforward to prove that *Î*^*cell*^ *= Î*, where *Î* is the mean scaled intensity for a population of cells.

**Fig 3:**
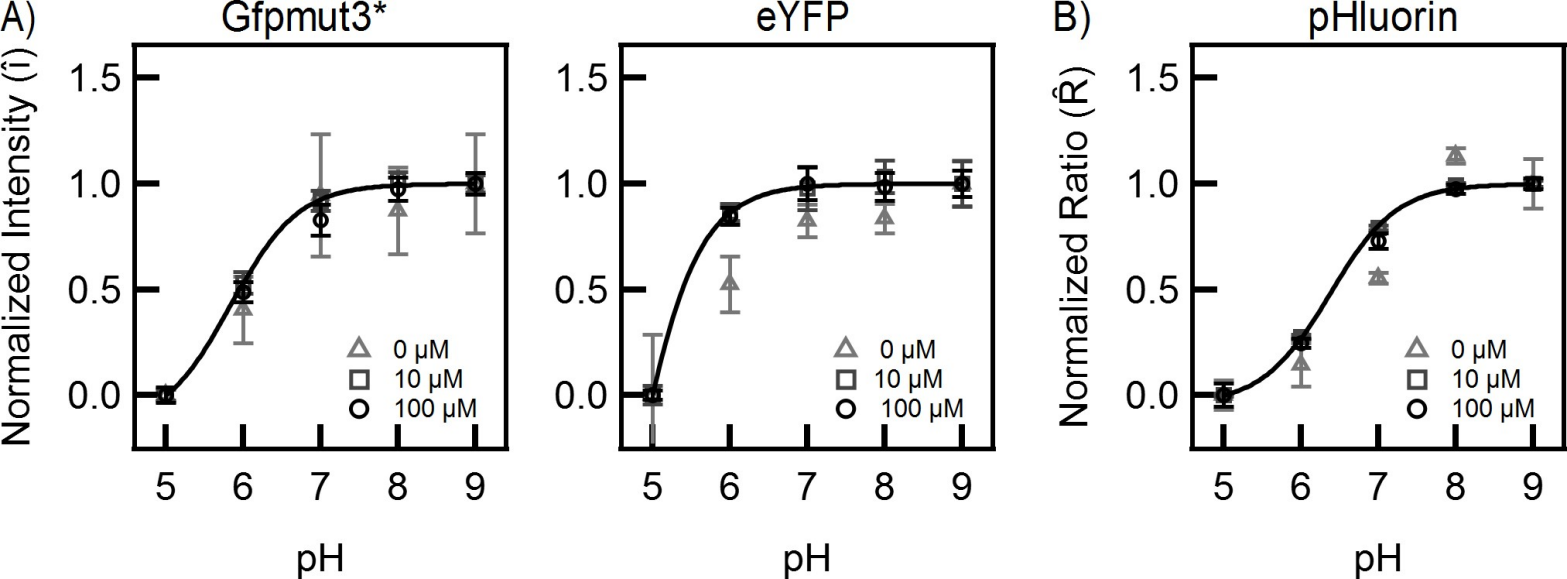
Normalized emission intensities and ratios as a function of cytoplasmic pH. **(A)** Normalized curves for Gfpmut3* (left panel) and eYFP (right panel) are indicated at different induction levels. The solid curves represent fits with Eq 5 with a single fit parameter, K. K = (1.8 ± 0.13)×10^3^ protons for Gfpmut3*; K = (2.9 ± 1.4)×10^4^ protons for eYFP. **(B)** The normalized emission ratios over a range of pH for pHluorin are indicated. The solid curve represents fits with Eq 5; K = 540 ± 24 protons.

Having demonstrated that the scaling analysis makes the signals from an intensity-based fluorophore independent of the absolute values of the fluorophore emissions and the expression levels, we tested it experimentally. According to the model, the scaling should cause the experimental curves to collapse onto standard curves for Gfpmut3* and eYFP. The equivalence between the mean normalized intensity of a population of cells and the normalized single-cell level output meant that we could simply scale the data in Fig 2 to test the prediction. Indeed, the normalized curves were superimposable as shown in Fig 3A, despite clear differences in the protein expression (S1 Fig) and illumination intensities (Fig 2). Eq 5 was then fitted to the experimental data with a single parameter, *K*, using Levenberg-Marquardt algorithm for iterative curve fitting (Igor Pro 7). For Gfpmut3*, a common fit was obtained since the normalized data at all expression levels coincided with each other. For eYFP, a good fit was obtained for the cases of the medium and high fluorophore expression levels (inducer IPTG concentrations of 10 and 100 μM). At low (basal) expression levels of eYFP, the experimental curve deviated from the standard curve. This was likely because of poor signal-noise ratio due to the paucity of fluorophores. The value of *K* for Gfpmut3* was an order of magnitude lower than that for eYFP. This is consistent with the higher pKa values reported for eYFP relative to some GFP variants [53].

### Dependence of ratiometric emission ratios on illumination and detection conditions

From Eq 1, the ratio of the emissions for a dual excitation fluorescent protein can be expressed as:

$$R(pH)=\frac{I_{prot}^{2}}{I_{prot}^{1}}\times \frac{{(N-N}_{prot})\delta_{2}+N_{prot}}{{(N-N}_{prot})\delta_{1}+N_{prot}}$$ (6)

A similar ratio can be written for the case of dual-emission proteins with minor modifications. The emission intensity of the deprotonated pHluorin is represented by $I_{unprot}^{1}$ at the higher excitation wavelength and $I_{unprot}^{2}$ at the lower excitation wavelength. The emission intensity of protonated pHluorin is represented by $I_{prot}^{1}$ at the higher excitation wavelength and $I_{prot}^{2}$ at the lower wavelength. Also, the relative intensities at the two wavelengths are: $\delta_{1}=\frac{I_{unprot}^{1}}{I_{prot}^{1}}$ and $\delta_{2}=\frac{I_{unprot}^{2}}{I_{prot}^{2}}$. The relation expectedly simplifies to an expression that is independent of the protein levels:

$$R(pH)=\frac{I_{prot}^{2}}{I_{prot}^{1}}\times \frac{\left({\left[H^{+}\right]}_{in}+K\delta_{2}\right)}{\left({\left[H^{+}\right]}_{in}+K\delta_{1}\right)}$$ (7)

Eq 7 suggests that the ratio R will be sensitive to changes in the pH only if *δ*_*2*_ *≠ δ*_*1*_. Thus, ratiometric proteins are only useful for pH measurements if the relative intensities at the two wavelengths are unequal. If *δ*_*2*_ and *δ*_*1*_ vary with illumination intensity and detector gain, then such dependencies are undesirable.

We explored whether a min-max normalization could make the ratios independent of the emission intensities for pHluorin. As shown in S2 Appendix, the scaling yielded an expression very similar to Eq 5:

$$\hat{R}(pH)=1-\frac{\left(1+\frac{K}{{\left[H^{+}\right]}_{in}^{pHmin}}\right)}{\left(1+\frac{K}{{\left[H^{+}\right]}_{in}}\right)}\beta$$ (8)

where $\beta =\frac{1}{1+\omega \frac{{\left[H^{+}\right]}_{in}}{\left(K+{\left[H^{+}\right]}_{in}\right)}}$ and $\omega =(\frac{1}{\delta_{1}}-1)$.

Since *β* depends on the relative emission intensities at the higher excitation wavelength, it is interesting to note that the experimental ratios for different expression levels collapsed on a single curve for pHluorin (Fig 3B). This suggests that the experimental $\hat{R}$ is only weakly dependent on *β*. Consistent with this notion, good fits were obtained for the normalized pHluorin emission ratios with Eq 5 (Fig 3B). The value of *K* for pHluorin was the lowest among the three fluorophores.

### Dynamic range and sensitivity of intensity-based probes

**Fig 4:**
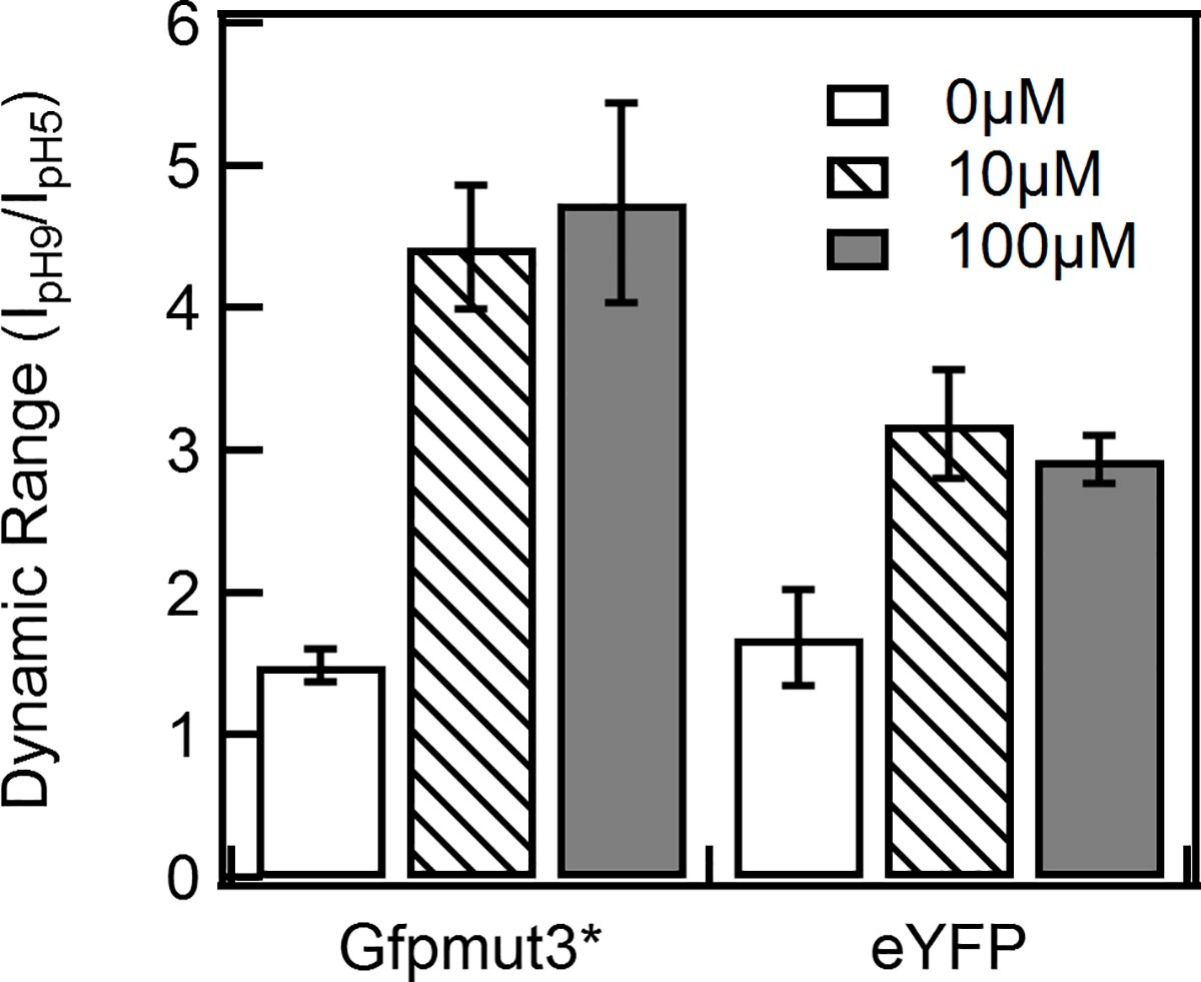
Dynamic range in pH measurements. Dynamic range–the ratio of the emission intensities at pH 9 (I_pH9_) and at pH 5 (I_pH5_)–are indicated for the intensity-based fluorophores. Induction levels are indicated in the figure legend; the difference in the means at 10 and 100 μM induction levels was not significant for either probes. Error bars were determined by error propagation.

The dynamic range for a pH-sensitive fluorophore is defined as the highest emission intensity divided by the lowest intensity [54]. It is a useful quantity in choosing an appropriate pH-sensitive fluorophore. For example, fluorophores with dynamic range ~ 1 are unsuitable probes due to low statistical power. The dynamic range was calculated for Gfpmut3* and eYFP at the three expression levels (Fig 4). The range was optimal for the medium expression and the high expression levels; it was the lowest at basal expression levels due to poor signal-to-noise ratios. Gfpmut3* exhibited a higher range than eYFP on our setup and for the strain of *E*. *coli* that we used, indicating higher accuracy for similar intrinsic noise.

**Fig 5:**
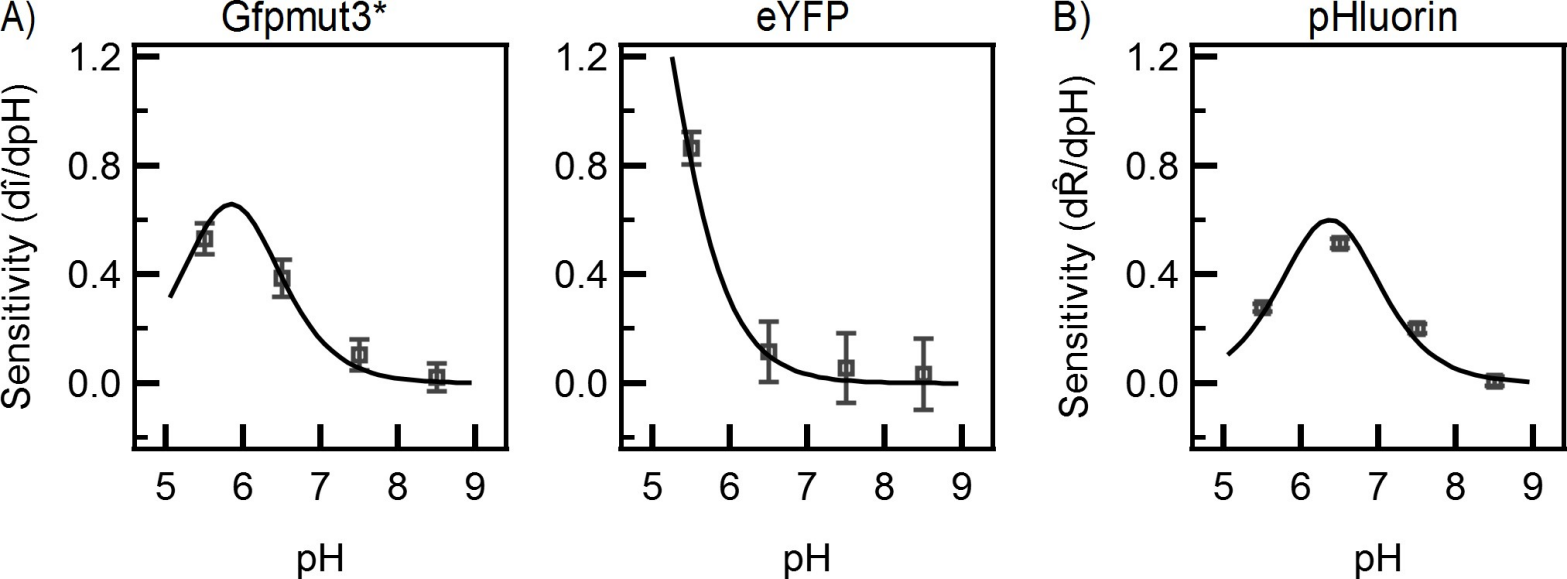
Fluorophore sensitivity versus pH. The experimental sensitivities (indicated by symbols) were calculated for the medium expression level case (10 μM IPTG) for each type of fluorescent protein. The solid lines indicate model predictions based on the K-values for the respective fluorophores (Fig 3). eYFP exhibited the highest sensitivity among the three fluorescent proteins. Gfpmut3* and pHluorin exhibited similar sensitivities and peaked between pH 6.0–6.5. Error bars were determined by error propagation.

Fluorophore sensitivity is another characteristic of interest when choosing fluorophores. Experimental sensitivities were calculated at the medium expression level (10 μM IPTG) since it provides a good dynamic range and is similar to the response at the high expression level (Fig 5). The predicted sensitivity curves were obtained by differentiating Eq 5. As shown in Fig 5, the experimental data and the predicted curves are in close agreement. Among the three fluorophores, eYFP is the most sensitive and exhibited high sensitivities at lower pH values (~5.0). Over physiological values of the intracellular pH though, eYFP had a low sensitivity. Thus eYFP is a suitable probe for the dynamics of protein self-assembly in live bacteria at physiological pH, since sensitivity to pH can confound results and interpretation [55–57]. Gfpmut3* and pHluorin exhibited similar sensitivities and the peaks in sensitivities occurred over a narrow range of pH (6.0–6.5).

## Discussions

We developed a two-state analytical model to interpret the pH-sensitive response of intensity-based and ratiometric fluorophores. We assumed two emission states for each fluorophore: a low intensity protonated state and a high intensity deprotonated state. We also assumed that the fluorophore concentration did not affect the proton abundances in the cell–the protonated fluorophore fraction was always in equilibrium with the cytoplasmic protons. The model suggested a simple scaling to render the emissions from intensity-based fluorescent proteins independent of the fluorophore expression levels. The predicted independence of the scaled signals was experimentally confirmed for Gfpmut3* and eYFP over a range of pH 5–9. The model was applicable for single cells as well as for a population.

The scaling analysis also suggested that the normalized signals from intensity-based fluorophores were independent of the absolute emission intensities of the protonated and deprotonated fluorophore. This is advantageous since it potentially eliminates the influence of experimental variabilities in illumination intensities, detector gain, and the differences in the optics used in different laboratories.

The value of *K* quantified the tendency of the fluorophore to protonate at a given pH. It shaped the emission curves, leading to a characteristic rise and plateauing observed for all three fluorescent proteins (Fig 2). The scaled signals from intensity-based probes were predicted to depend solely on the parameter *K* (Eq 5), which is independent of the fluorophore concentration and the acquisition setup. Thus, despite the non-linearity of the scaled response curve, the absolute internal pH can be determined from an intensity measurement with just two calibrated values: the minimum intensity (at pH 5) and the maximum intensity (at pH 9). If the fluorophore levels are anticipated to vary between two strains for some reason (or between other types of treatments), then the minimum and maximum intensities must be measured for each strain (or treatment). Since fluorophore levels are unlikely to vary significantly over a few seconds, our approach is likely to prove valuable in determining changes in absolute pH values from dynamic response measurements (Fig 1). The value of *K* was ~ 20 times higher for eYFP compared to pHluorin in the strains that we employed here. In addition to depending on the chromophore type and the bacterial species, *K* is likely to be impacted by environmental stressors and cell metabolism.

We extended the model to analyze the emissions from ratiometric probes. The emission ratio was sensitive to variations in the relative emission intensities of the fluorophores—simple scaling could not eliminate this dependence. Therefore, it is best to maintain the same illumination/detection conditions over which prior calibrations have been performed. The sensitivity of the emission ratios to the pH was predicted to be due to the difference between *δ*_*1*_ and *δ*_*2*_: these are the ratios of the emission intensities of the deprotonated and protonated fluorophore at the two excitation wavelengths (Eq 7). For *δ*_*1*_ = *δ*_*2*_, the emission ratios will be insensitive to pH variations. This provides an important criterion when designing ratiometric probes.

The suitability of a probe for pH detection is determined by several factors including the sensitivity to pH fluctuations, the intrinsic noise in its response, and the dynamic range. eYFP exhibited the highest sensitivity (at pH ~ 5) and dropped sharply at higher pH values. Thus, in the strains employed in this work, eYFP is a suitable probe under acidic conditions. Since Gfpmut3* and pHluorin exhibited similar sensitivities that peaked around ~ pH 6.0, the latter is more reliable in detecting small changes in the pH due to the lower noise associated with its emission ratios. Gfpmut3* displayed a higher dynamic range than eYFP, which translates to a superior signal-to-noise ratio. Induction from the *pTrc99A* vector with 10 μM IPTG was determined to be optimal since it provided the best dynamic range, sensitivities and signal-noise ratios. Higher expression levels of the chimeric proteins may cause unwanted effects such as the formation of inclusion bodies and increased toxicity [58] and thus, should be avoided. In the absence of the inducer, the basal expression proved too low to obtain reliable measurements [59].

Several types of intensity-based as well as ratiometric fluorophores are available that can be excited at higher wavelengths to avoid the blue light effect [32, 38–40]. However, intensity-based fluorophores require simpler setups (single wavelength excitation and emission), which enables rapid sampling of short-time dynamics (Fig 1). We anticipate that our analysis and experiments will help extend the applications of intensity-based fluorophores for pH-sensitive measurements, and provide a useful guide for selecting and using appropriate fluorescent probes.

## Supporting information

S1 Fig Relative expression levels of Gfpmut3* at different IPTG concentrations. Absolute intensities in live cells were obtained over three induction levels of Gfpmut3*. The same illumination intensities and detection settings were used for all three datasets. Mean values are from four technical replicates; standard error is indicated. Differences in intensities between 0 and 10 μM, and between 10 and 100 μM were statistically significant (p-value < 0.05).
(TIF)

S2 Fig Emissions from pH-insensitive DsRed-Express. DsRed-Express was expressed with 100 μM IPTG. Each mean value was calculated from four technical and three biological replicates. Standard error is indicated based on the biological replicates. A two-tailed, paired t-test was performed to compare each adjacent pair. Differences in means were insignificant (p-value > 0.05).
(TIF)

S1 Appendix (DOCX)

S2 Appendix (DOCX)
