# Supplementary figures and images for: Protein expression-independent response of intensity-based pH-sensitive fluorophores in Escherichia coli
Source: PLoS One. 2020 Jun 18;15(6):e0234849. doi: 10.1371/journal.pone.0234849 (PMC7302705; doi:10.1371/journal.pone.0234849)

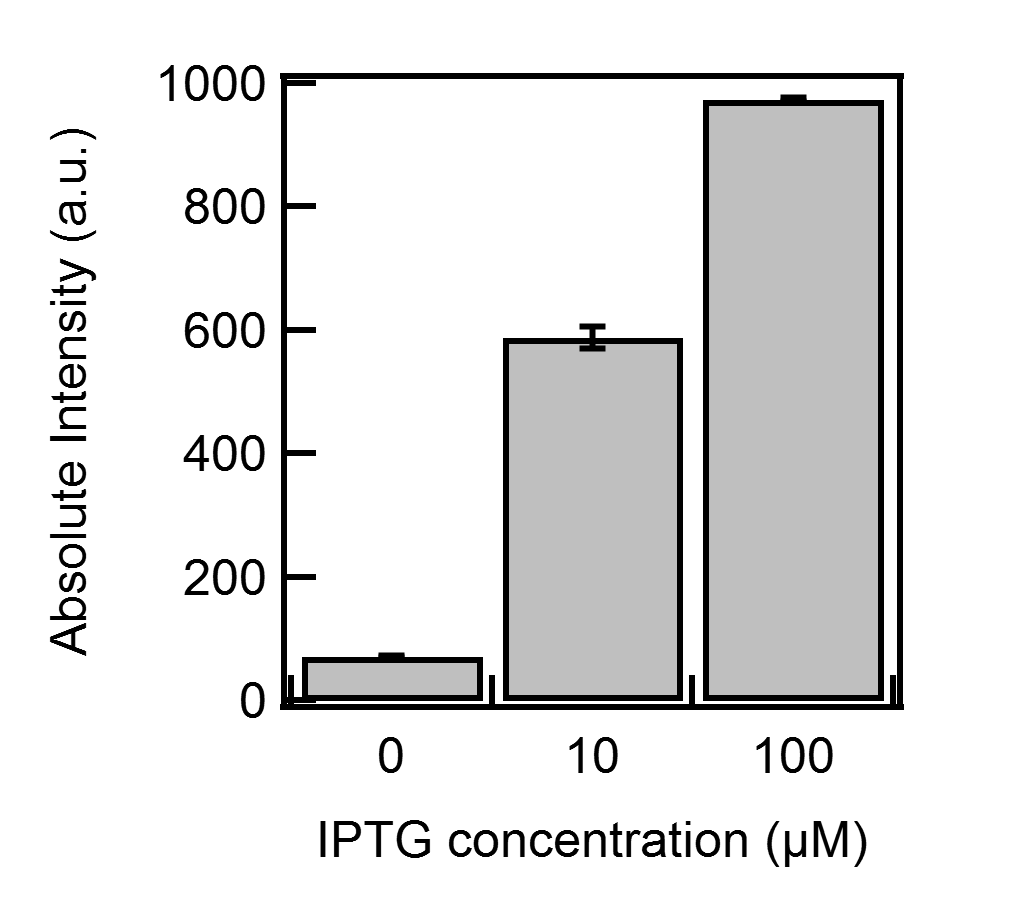

Supplement: S1 Fig — Absolute intensities in live cells were obtained over three induction levels of Gfpmut3*. The same illumination intensities and detection settings were used for all three datasets. Mean values are from four technical replicates; standard error is indicated. Differences in intensities between 0 and 10 μM, and between 10 and 100 μM were statistically significant (p-value < 0.05). (TIF) [file pone.0234849.s001.tif]

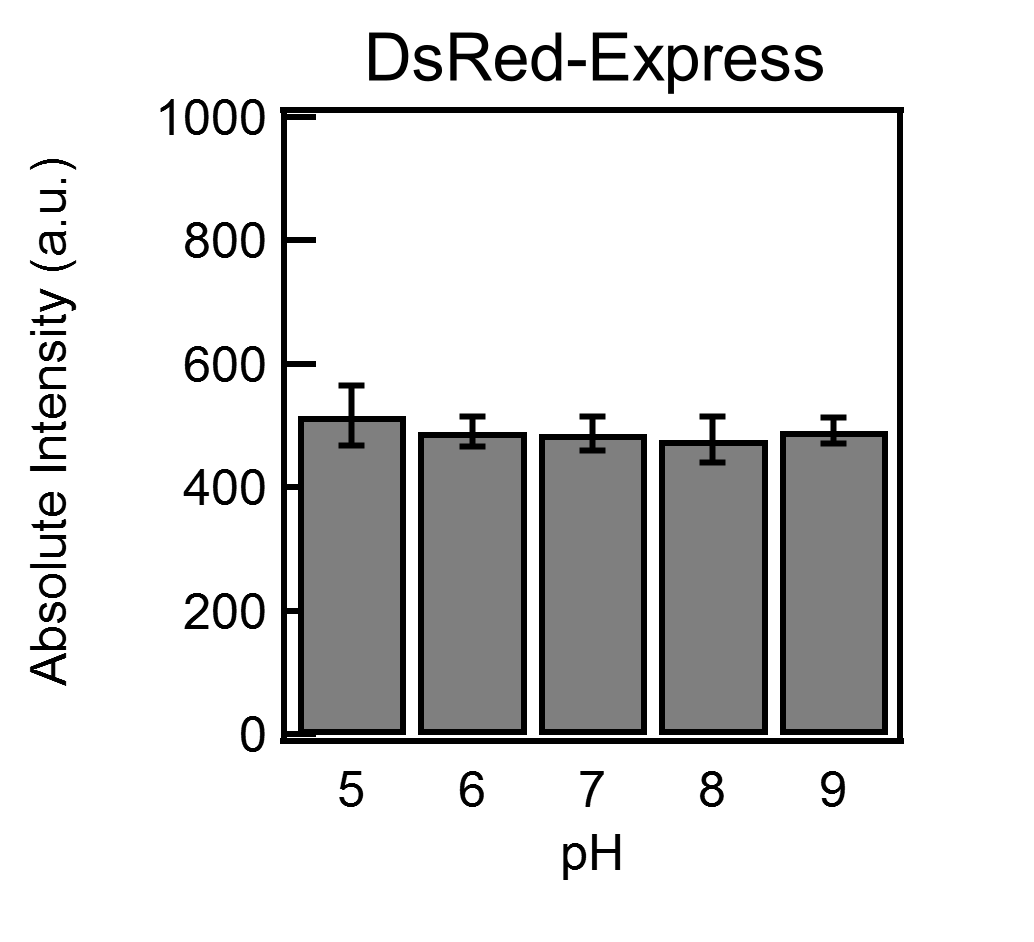

Supplement: S2 Fig — DsRed-Express was expressed with 100 μM IPTG. Each mean value was calculated from four technical and three biological replicates. Standard error is indicated based on the biological replicates. A two-tailed, paired t-test was performed to compare each adjacent pair. Differences in means were insignificant (p-value > 0.05). (TIF) [file pone.0234849.s002.tif]
