# Supplementary material for: Protein expression-independent response of intensity-based pH-sensitive fluorophores in Escherichia coli
Source: PLoS One. 2020 Jun 18;15(6):e0234849. doi: 10.1371/journal.pone.0234849 (PMC7302705; doi:10.1371/journal.pone.0234849)
Supplement: S1 Appendix — (DOCX) [file pone.0234849.s003.docx]

# S1 Appendix

The maximum and minimum emission intensities are defined at pH_max_ and pH_min_, which indicate the range of experimental conditions:

$I_{max}^{cell}=N_{prot}^{pH max}I_{prot}+(N-N_{prot}^{pH max})I_{unprot}$ (a)

$I_{min}^{cell}=N_{prot}^{pH min}I_{prot}+(N-N_{prot}^{pH min})I_{unprot}$ (b)

$$\Delta I=I_{max}^{cell}-I_{min}^{cell}$$

$=\left( N_{prot}^{pH min}-N_{prot}^{pH max} \right)I_{prot}(\delta-1 )$ c)

where, $\delta=I_{unprot}/I_{prot}$

$$\hat{I}^{cell}=\frac{I^{cell}-I_{min}^{cell}}{\Delta I}$$

$=\left( N_{prot}^{pH min}-N_{prot}^{pH} \right)/\left( N_{prot}^{pH min}-N_{prot}^{pH max} \right)$ (d)

When *pH min* and *pH max* are separated by several orders of magnitude, the expression reduces to:

$=\left( 1-N_{prot}^{pH}/N_{prot}^{pH min} \right)$ (e)

Thus, the normalized intensities $\hat{I}\left( pH \right)$ are independent of the differences in the absolute intensities of the protonated and the deprotonated states of the fluorophore.
