# Supplementary material for: Protein expression-independent response of intensity-based pH-sensitive fluorophores in Escherichia coli
Source: PLoS One. 2020 Jun 18;15(6):e0234849. doi: 10.1371/journal.pone.0234849 (PMC7302705; doi:10.1371/journal.pone.0234849)
Supplement: S2 Appendix — (DOCX) [file pone.0234849.s004.docx]

# S2 Appendix

$R\left( pH \right)=\frac{I_{prot}^{2}}{I_{prot}^{1}}\times\frac{{(N-N}_{prot})\delta_{2}+N_{prot}}{{(N-N}_{prot})\delta_{1}+N_{prot}}$ (f)

$\hat{R}\left( pH \right)=\frac{R\left( pH \right)-R_{min}}{R_{max}-R_{min}}$

$=\frac{\left( N_{prot}^{pH min}-N_{prot}^{pH} \right)}{\left( N_{prot}^{pH min}-N_{prot}^{pH max} \right)}\frac{(N-N_{prot}^{pH max})\delta_{1}+N_{prot}^{pHmax}}{{(N-N}_{prot})\delta_{1}+N_{prot}}$ (g)

$\left[ H^{+} \right]_{in}^{pH max=9}\sim1$, ∴ $N_{prot}^{pH max}\ll N$,

$\hat{R}\left( pH \right)\sim\frac{\left( N_{prot}^{pH min}-N_{prot}^{pH} \right)}{\left( N_{prot}^{pH min}-N_{prot}^{pH max} \right)}\frac{N\delta_{1}}{{(N-N}_{prot})\delta_{1}+N_{prot}}$

$\to\delta_{1}=\frac{I_{unprot}^{1}}{I_{prot}^{1}}$ (h)

$\hat{R}\left( pH \right)\sim\frac{\left( N_{prot}^{pH min}-N_{prot}^{pH} \right)}{\left( N_{prot}^{pH min}-N_{prot}^{pH max} \right)}\frac{1}{1+N_{prot}(1/\delta_{1}-1)/N}$ (i)

$\hat{R}\left( pH \right)\sim\frac{\left( N_{prot}^{pH min}-N_{prot}^{pH} \right)}{\left( N_{prot}^{pH min}-N_{prot}^{pH max} \right)}\frac{1}{1+\frac{K\left[ H^{+} \right]_{in}}{\left( K+\left[ H^{+} \right]_{in} \right)}\omega}$ (j)

where, $\omega= \frac{1}{N}\left( \frac{1}{\delta_{1}}-1 \right)$.
